# WH Craib: a critical account of his work

**Published:** 2009-02

**Authors:** DP Naidoo

**Affiliations:** Department of Cardiology, University of KwaZulu-Natal, Inkosi Albert Luthuli Central Hospital, Durban

## Abstract

**Summary:**

One hundred years after its introduction, the ECG remains the most commonly used cardiovascular laboratory procedure. It fulfils all the requirements of a diagnostic test: it is non-invasive, simple to record, highly reproducible and can be applied serially. It is the first laboratory test to be performed in a patient with chest pain, syncope or cardiac arrhythmias. It is also a prognostic tool that aids in risk stratification and clinical management.

Among the many South Africans who have made remarkable contributions in the field of electrocardiography, Don Craib was the first to investigate the changing patterns of the ECG action potential in isolated skeletal muscle strips under varying conditions. It was during his stay at Johns Hopkins Hospital in Baltimore and Sir Thomas Lewis laboratory in London that Craib made singular observations about the fundamental origins of electrical signals in the skeletal muscle, and from these developed his hypothesis on the generation of the action potential in the electrocardiogram. His proposals went contrary to scientific opinion at the time and he was rebuffed by the scientific community. Frank Wilson subsequently went on to develop Craib’s doublet hypothesis into the dipole theory, acknowledging Craib’s work.

Today the dipole theory is fundamental to the understanding of the spread of electrical activation in the myocardium and the genesis of the action potential.

**W H CRAIB (1895–1982)**

In rebus scientiarum auctoritatem dubitate

[in matters of science doubt authority]^1^

Bothwell

## Summary

Don Craib was born in 1895 and grew up in the Karoo. After coming first in the Cape matriculation examination in 1911, he graduated with honours in 1914, majoring in mathematics, applied mathematics and physics at the South African College (now the University of Cape Town). Well known for his introduction of the doublet theory to the understanding of the ECG and his subsequent arguments with Einthoven and Lewis, Craib was responsible for the early experiments explaining the generation of the action potential in the ECG as we understand it today.

In the midst of his studies he was called up to serve against Germany in the German–South West African Campaign. Following that, he served as a trench mortar officer in a British division in France, when he received the Military Cross for valour in World War I. After the war he took up medical studies and qualified in medicine at Guy’s Hospital in London. As a registrar in the medical wards there he showed great intellectual prowess and was awarded the prestigious Rockefeller Fellowship to Johns Hopkins Hospital, Baltimore where he studied the ECG action potentials recorded on isolated animal heart muscle. He found that the descriptions of the generation of the action potential by the doyens of ECG at the time, namely Einthoven^2^ and Sir Thomas Lewis,^3^ were contrary to his understanding of electricity. Forbidden to continue with his work at Hopkins, he returned to England to work in the laboratory of Sir Thomas Lewis. He published his experiments in the new journal, *Heart*,^4^ edited by Lewis, and subsequently published a treatise on the electrocardiogram under the aegis of the Medical Research Council, London.^5^

Sadly, history repeated itself and he left after a fall-out with Lewis. It is at this time that he made a life-changing decision in his career, which took him away from research, and into the bind of clinical medicine. ‘Unable to accept the dogmas and disciplines of scientific authority’,^1^ he returned to South Africa and took up a part-time post as professor of medicine at the University of the Witwatersrand in 1932. When Craib took over the department from Prof OK Williamson in 1932, he found that he was expected to care for patients, teach students, as well as undertake some research on a meager salary of £750 per annum.^1^ With minimal staff to assist him, he spent most of his time at the bedside, endeared himself to his students and colleagues, and established himself as a dominant figure in South African clinical medicine.^6^

Not surprisingly, Craib performed little research during this period, and furthermore, between 1939 and 1946 he served as physician consultant to the South African Forces during World War II. Shortly upon his return to medical school, he had a disagreement with the Senate, resigned in 1947, and took up private practice in Port Elizabeth for the next 15 years.

‘What a mistake Wits University made when they allowed him to go!’.^7^ Craib’s leaving catapulted the university into transformation and led to the development of a formal departmental structure. He should have applied for the full-time chair that was subsequently created. Prof Andries Brink points out that in 1950 he was training as internist at the University of Pretoria, when a dispute arose between the registrars in training and the new head of the department. The latter made unacceptable demands for certain aspects of patient treatment. The dispute landed up in court and Craib was invited to be a medical assessor for the presiding judge. After the findings, a new head was appointed and the University accepted costs. Craib was also the external examiner for Andries Brink’s MD thesis (pers commun).

In the 1960s, Craib was invited to join the Council for Scientific and Industrial Research (CSIR), which allocated funds for medical research and there he served as an advisor working once again in the field of medical research. In his book on Craib, EB Adams says that Craib claimed this to be the happiest period of his life.^6^ Craib was appointed as vice president (1963–1968) of the Chairmanship of the Committee for Research in Medical Science (CRMS) (jocularly named CRUMBS), and Craib supported the appointment of Andries Brink to succeed him, with whom he subsequently developed a fairly long and close relationship. In 1969 Brink became the first president of the South African Medical Research Council.

Craib then retired to his home village of Somerset East, where he grew up, not getting the satisfaction of the full recognition that he so rightly deserved for the immenseness of his work on the doublet hypothesis, until much later in life.

## Craib – the man

Few people alive today have had the good fortune of meeting this wondrous man; ‘liberal in the Hofmeyr tradition, he behaved the same way with everyone – white, black or coloured’. He was a gifted teacher and warm person, at the same time unpredictable, and intolerant of sloppy thinking. He was a generous man and charged little, often seeing patients for nothing.^7^ He founded the Craib prize at the University of the Witwatersrand, which was awarded to the top non-European finalist in the MB BCh examinations. From Perth, Krish Somers writes:^8^ ‘as far as I can recall, I was the second awardee of the Craib prize in 1949. The princely sum of £5.00 was most welcome at the start of my internship, the first intern ever at St Aidan’s Hospital in Centenary Road where I unlearnt medicine! Years later, because of the designation, “top non-European student” smacked of apartheid in the awarding of prizes, it is my understanding that non-European students at the University of the Witwatersrand decided to boycott the prize. I wonder what happened to the money that Craib had endowed to Wits Medical School.’

It is interesting to note that Craib also established a Craib prize in the Department of Medicine of the then University of Natal to the student who made the best academic progress from the fourth to the final year (YK Seedat, pers commun). Seedat points out that he had ‘many communications with Professor Craib over the concept that beta-blockers could be used in the treatment of hypertension; a concept that Craib would not accept in the 1970s.’

Craib was greatly revered at home and abroad for his contributions to medicine, and much loved by a wide circle of family and friends and, in the words of his students, ‘he was always questioning, he was one of a kind’.^7^,^9^ In his account, Bothwell remarks on the appropriateness of the inscription on a bowl that was presented to Craib when he left the CSIR (see title inscription above).

Craib was relentless in his pursuit of the truth. The title of EB Adam’s book, *In Search of Truth: A Portrait of Don Craib* reflects Craib’s philosophy.^9^ Indeed, a typical Craib question is demonstrated in his correspondence to one of his colleagues: ‘Please do me a favour! Just send me a postcard with what in your opinion is an example of “Truth” that is not at the same time a fact, a reality, an event. Scientific literature abounds in the use of the word!’^7^

## Craib’s research

Craib had a clarity of thought that enabled him to challenge authority in an incisive manner, an attribute that at the same time led to his fall-out with the scientific community. After Sir Thomas Lewis published his work describing the ‘circus movement’ in the maintenance of atrial tachyarrhythmias, the interest shifted from arrhythmias to the theory of the ECG, to ECG leads and abnormalities of waveforms. In 1927 Craib published his seminal work in *Heart*, which was contrary to the beliefs of both Einthoven and Lewis at the time.^4^

From his experiments, he formulated his doublet hypothesis, referring to the wave of excitation that passes down the muscle tissue across a series of interfaces as a succession of doublets, each of which is so arranged that the positive pole is a short distance ahead of the negative pole. He coined the term ‘front’ to describe the direction in which the wave is travelling. His views challenged the current thinking at the time, that described a general ‘negativity of the active region’ when muscle tissue is stimulated.

Following this, Lewis’ student, Frank Wilson, who had heard Craib’s presentation at a meeting, went on to develop the doublet hypothesis into the dipole theory as we know it today. In 1934, Wilson described the unipolar leads having an exploring and indifferent electrode. The concept included a central electrode close to null potential (a combination of the three unipolar limb leads) and an exploring electrode placed on selected areas of the body. Wilson also used the exploring electrode as a chest lead to follow the course of acute infarction.^10^ In 1944, Wilson and associates published an article titled, ‘The precordial electrocardiogram’ describing the utility and contribution of the unipolar precordial leads to clinical cardiology.^11^

Craib’s simple experiments showed that it was not necessary to consider individual cardiac muscle cells, as was Einthoven’s fundamental postulate, in understanding the action potential. He considered the myocardium a syncytium of extremely short individual fibres in which electrical activation proceeded in wave fronts from negative to positive. Craib showed that the diphasic action potential recorded from isolated muscle strips is a composite of forces produced by depolarisation and repolarisation. ‘Using immersed heart muscle strips (*to simulate tissues immersed in an extensive conducting medium*) a simple diphasic response was never obtained; the curves were always polyphasic, and the initial deflection invariably indicated relative positivity of the proximal contact’.^12^

In so doing, he disproved the negativity hypothesis, showing that the electrical potential varied from positive to negative and back to positive as the action potential wave front progressed. Craib showed that the field of potential due to such currents could best be explained on the basis of doublets.^12^ By showing that in relation to ground (zero) potential, the ECG action potential was recorded as deflections above and below the baseline during each heartbeat, he invalidated the statements made by Lewis in 1924,^3^ and still perpetuated by others.^13^ Craib pursued similar experiments in the years that followed^14^-^16^ when he discovered that the writers still referred to the ‘negativity hypothesis’ which he had so conclusively disproven in 1927.

In a recent article,^17^ Nelson *et al.* endorse Craib’s contention when they describe the heart as electrically consisting of a large number of dipole generators during excitation or recovery. These individual dipoles summate into one or more ‘effective’ dipoles, where an effective dipole is one which is evident at the body surface.^4^ Several factors affect the dipole potential, among them being heart weight or body weight and tissue inhomogeneity.^17^,^18^ This may be explained by the increase in total current flow with increased cardiac mass. Since in a large heart there are more current generators, one would expect a greater current density at the body surface. It is the application of this logic that has enabled our understanding of the effects that ventricular hypertrophy and myocardial injury have on the spread of the excitation within the ventricles, and the subsequent changes in the action potentials on the electrocardiogram.

## Craib’s vindication

Craib must have felt a deep sense of failure and rejection when he returned home, accused of plagiarising the doublet hypothesis from Einthoven, who had in fact opposed it, until it gained acceptance following the publication of Wilson’s dipole theory. As a result, his grants from the Rockefeller foundation were withdrawn. It was only in 1976, after Pruitt’s revelations in the *John Hopkins Journal*,^19^ and after Leo Schamroth delivered a series of brilliant lectures, that Craib was totally vindicated, having received an apology from the foundation and been reinstated on the Johns Hopkins roll of honour.^20^

It is indeed noteworthy that in 1938 Macleod had placed Craib’s work in perspective, giving an account of the several theories, beginning with the negativity hypothesis and ending with the work of Wilson. Craib is credited with first propounding the theory of dipolar electrocardiography in the early 1920s.^21^ He received further recognition for his work when, in 1964, George Burch and De Pasquale^22^ published *History of the ECG*. In chapter III of Burch’s book, titled *Great Men of Electrocardiography*, the name WH Craib stands proudly among Einthoven, Lewis, Wilson and several others, as one of the great men who revolutionised the field of electrocardiography.

## Conclusion

Don Craib died in 1982, just over 25 years ago, aged 86, having made an indelible impression not just in the world of electrocardiography, but also on his fellow colleagues and students. In the words of Tom Bothwell, ‘Why do I and my contemporaries remember a couple of lectures so vividly? It is because it was the first time that we had been invited to think for ourselves and question authority?’^1^

It has been said that towards the latter part of his life, Craib had a sense of self-doubt and failure. His sister could not have put it better when in a letter to him she said ‘… you would of course, have been a very great figure in the medical research world had you stayed in research’.^1^

In Fisch’s centennial historical review of the ECG, I am pleased to say that Craib has rightfully earned his spot as one of the great men who helped shape electrocardiography as it is today.^23^
